# Spectroscopic and Photophysical Investigation of Model Dipyrroles Common to Bilins: Exploring Natural Design for Steering Torsion to Divergent Functions

**DOI:** 10.3389/fchem.2021.628852

**Published:** 2021-02-17

**Authors:** Clayton F. Staheli, Jaxon Barney, Taime R. Clark, Maxwell Bowles, Bridger Jeppesen, Daniel G. Oblinsky, Mackay B. Steffensen, Jacob C. Dean

**Affiliations:** ^1^ Department of Physical Science, Southern Utah University, Cedar City, UT, United States; ^2^ Department of Chemistry, The Pennsylvania State University, State College, PA, United States; ^3^ Department of Chemistry, North Carolina State University, Raleigh, NC, United States; ^4^ Department of Chemistry, Princeton University, Princeton, NJ, United States

**Keywords:** torsional deactivation, Z-E isomerization, light harvesting, photoreception, Bilins, Dipyrroles, Phycobiliproteins

## Abstract

Biliproteins are a unique class of photosynthetic proteins in their diverse, and at times, divergent biophysical function. The two contexts of photosynthetic light harvesting and photoreception demonstrate characteristically opposite criteria for success, with light harvesting demanding structurally-rigid chromophores which minimize excitation quenching, and photoreception requiring structural flexibility to enable conformational isomerization. The functional plasticity borne out in these two biological contexts is a consequence of the structural plasticity of the pigments utilized by biliproteins―linear tetrapyrroles, or bilins. In this work, the intrinsic flexibility of the bilin framework is investigated in a bottom-up fashion by reducing the active nuclear degrees of freedom through model dipyrrole subunits of the bilin core and terminus free of external protein interactions. Steady-state spectroscopy was carried out on the dipyrrole (DPY) and dipyrrinone (DPN) subunits free in solution to characterize their intrinsic spectroscopic properties including absorption strengths and nonradiative activity. Transient absorption (TA) spectroscopy was utilized to determine the mechanism and kinetics of nonradiative decay of the dipyrrole subunits, revealing dynamics dominated by rapid internal conversion with some *Z*→*E* isomerization observable in DPY. Computational analysis of the ground state conformational landscapes indicates enhanced complexity in the asymmetric terminal subunit, and the prediction was confirmed by heterogeneity of species and kinetics observed in TA. Taken together, the large oscillator strengths (*f* ∼ 0.6) of the dipyrrolic derivatives and chemically-efficient spectral tunability seen through the ∼100 nm difference in absorption spectra, validate Nature's "selection" of multi-pyrrole pigments for light capture applications. However, the rapid deactivation of the excited state via their natural torsional activity when free in solution would limit their effective biological function. Comparison with phytochrome and phycocyanin 645 crystal structures reveals binding motifs within the *in vivo* bilin environment that help to facilitate or inhibit specific inter-pyrrole twisting vital for protein operation.

## Introduction

The light harvesting process in photosynthesis constitutes the initial step that triggers the subsequent chain of electron transfer events and chemical reactions. It involves first the absorption of light by a chromophore, or pigment, followed by the transport of that energy to a reaction center site where it is finally converted into a charge separation. This process typically occurs at efficiencies approaching unity, despite the tens to hundreds of pigment-pigment energy transfer events required to span the spatial extent of the light-harvesting apparatus (Glazer, 1989; MacColl, 1998; Wientjes et al., 2013; Mirkovic et al., 2017). Such remarkable efficiencies are accomplished by utilization of antenna proteins that augment reaction centers for dramatically enhanced light capture, while simultaneously providing a pigment-coupling network to facilitate rapid and directional excitation transport prior to quenching processes. These features are realized by elegant molecular design at the single antenna-protein level where a specific arrangement of pigments, in orientations and positions enforced by local interactions with the surrounding protein scaffold, lead to a collectively optimized spectral and energetic landscape of the pigment network as a whole.

**FIGURE 1:**
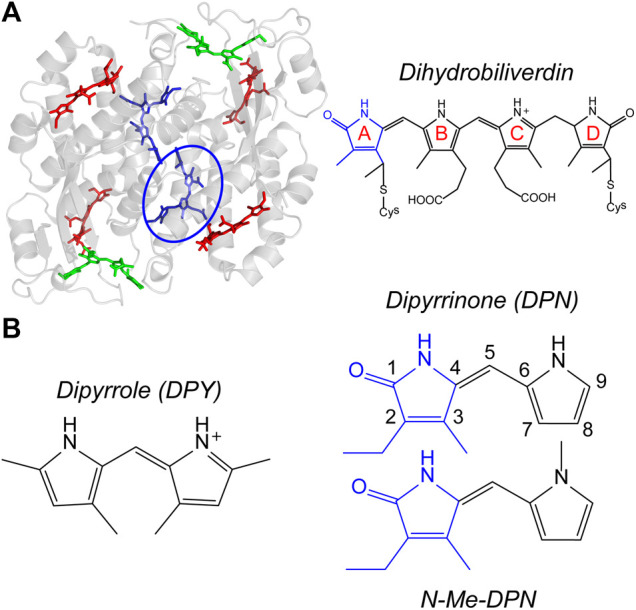
**(A)** PC645 crystal structure with bilin types color-coded, and chemical structure of dihydrobiliverdin (DBV) referenced by the blue circle. **(B)** Chemical structures of dipyrroles studied in this work.

Phycobiliproteins are light harvesting antennas utilized by cyanobacteria and red algae in the form of phycobilisome supermolecular complexes, and also by cryptophyte algae where they are individually suspended in the thylakoid lumen (Glazer and Wedemayer, 1995; Scholes et al., 2012). These proteins are unique in their characteristically different, yet variable light capture properties compared to chlorophyll-based light harvesting proteins such as LHCII, chlorosomes, and the photosystems. Typically, their absorptions occur in the region between the prominent Soret and *Q*_y_ bands of chlorophyll and red of the primary carotenoid absorptions, enabling the capture of light filtered through Chl-based photosynthetic organisms. They achieve this targeted light capture by employing linear tetrapyrrole pigments called bilins, enabling significant freedom to structural and therefore spectral properties of their light harvesting machinery. That tunability in light capture is granted specifically by variable pigment conjugation, local contortions of the bilin geometry imposed by particular interactions with the protein scaffold, and finally the spatial configuration of neighboring pigments. These features taken together also imparts rapid and efficient energy transfer/funneling throughout and between light harvesting complexes. The light-harvesting strategy of cryptophyte algae involves suspending single phycobiliproteins in the lumen, each with a set of different bilin types to allow for extensive energy funneling within a single protein. A model example is the phycocyanin 645 (PC645) protein shown in Figure 1A, where specific bilin types are color-coded. These proteins all have a (αβ)_2_-type structure with pseudo-twofold symmetry between each αβ monomer. The β subunit binds three bilins, while the extended polypeptide α subunit binds a single bilin which typically lies near the center of the protein (Harrop et al., 2014). The types of bilins differ in their extent of conjugation as well as the number of covalent linkages to the apoprotein, with either one linkage at the A ring or two at rings A and D (Figure 1A, right) (Glazer, 1985; Glazer, 1989; MacColl, 1998; Harrop et al., 2014). Among the various cryptophyte phycobiliproteins sequenced and whose crystal structures have been solved, significant homology is demonstrated both in sequence and αβ conformation (Harrop et al., 2014), showing that light capture differences occurs predominantly by exchanging bilin types.

While the comparatively flexible, linear bilin structure is advantageous for bestowing spectral tunability to the organism, this same feature also introduces the possibility for deactivation pathways which are parasitic to light harvesting via “twisting” motions about the respective methine bonds separating pyrrolic rings. Generally, the torsional freedom about bonds separating two parts of a conjugated system, as in the case of bilins, will lead to nonradiative deactivation of the excited molecule through direct internal conversion or by isomerization through a conical intersection. The latter of which is a common pathway utilized in nature to initiate signaling through photoreception. One example of this is the initial step in vision where the retinal molecule isomerizes in less than 100 fs following photon absorption (Wang et al., 1994; Polli et al., 2010), leading to a conformational change in the rhodopsin protein followed by a transduction cascade (Palczewski, 2012; Molday and Moritz, 2015). Perhaps more relevant to bilin light harvesting however, are photosensory phytochromes and cyanobacteriochromes found in plants, bacteria, cyanobacteria, and fungi which incorporate a single bilin photoswitch capable of undergoing isomerization following light absorption. This behavior ultimately results in downstream photomorphogenesis among other dynamical processes vital for growth and survival (Rockwell et al., 2006; Ulijasz and Vierstra, 2011). In canonical phytochromes, the isomerization event takes the red-absorbing species (P_r_) to the Lumi-R intermediate state, before completing the process to the far-red absorbing meta-stable P_fr_ form. The process primarily involves rotation of the D pyrrole ring from a nominally *Z* conformation (about the nearest methine double bond) to *E*, coincidentally with local protein adjustments in the binding pocket to promote the transformation and stabilization of each state. The reaction occurs typically with a quantum yield of ∼0.15 (Lamparter et al., 1997; Dasgupta et al., 2009), and it is both thermally and photochemically reversible. The remaining ∼85% of the phytochrome population reverts back to the ground state in 3 ps via internal conversion, apparently outcompeting the isomerization pathway (Dasgupta et al., 2009).

A fascinatingly stark contrast is noted between the two biological contexts/functions utilizing bilins. Torsional dynamics between pyrrole rings must be eliminated in photosynthetic light harvesting to retain the electronic excitation long enough for many energy transfer events. While that torsional freedom is supported and controlled in the photoreception mechanism of phytochromes and cyanobacteriochromes. In both contexts however, the local pigment-protein and water interactions appear crucial to confer bilin function in a controllable fashion. Here, we seek to characterize the innate torsional freedom and conformational landscape of bilins absent explicit protein interactions. In this way, the importance and extent of individual protein/water contacts in facilitating the functionality of bilins can be assessed from an appropriate reference point―the free molecule in solution.

In order to do this in a bottom-up fashion however, the tetrapyrrole structure (incorporating three methine bridges with six separate torsional coordinates) must be broken down into dipyrrolic subunits which incorporate only a single methine group with two torsional degrees of freedom, thereby simplifying the nuclear degrees of freedom responsible for relevant excited state dynamics. In doing so, one finds two unique subunits representative of either the terminal pyrrolinone-pyrrole pair (dipyrrinone, DPN), or the central dipyrrole pair (DPY) devoid of the carbonyl group.

While little has been reported on the spectroscopic properties of the dipyrrole “core” subunit specifically, it is a spectroscopic analogue to a common marine cyanobacterial light-harvesting bilin called phycourobilin (PUB). In the case of the PUB pigment, the electronic conjugation extends only over the central B and C rings which are subsequently bound to the terminal pyrrolinone rings via methylene groups (i.e. single bonds). Incorporating a bilin with such a restricted conjugation length enables the organism to efficiently capture light further into the blue-green region, which is an ecological requirement for these organisms to do photosynthesis in subsurface oceanic waters where the penetration depth of blue light is best. Given this property, it has been suggested that PUB is the most abundant phycobilin in the ocean (Blot et al., 2009). Since the dipyrrole core of PUB is responsible for the relevant visible absorption, at least a cursory comparison of its spectroscopic properties can be made to the model DPY subunit studied in this work.

There has been interest in dipyrrinone since the discovery of phototherapy of neonatal jaundice through photochemistry of bilirubin. The bilirubin structure is effectively two dipyrrinone halves separated by a methylene bridge, and the operative mechanism responsible for its photochemistry is in fact *Z*→*E* isomerization at the D pyrrole ring with a quantum yield of ∼0.1 (Mazzoni et al., 2003), with the remainder of the excited population decaying back to the ground state (∼15 ps) similar to the photochemistry of phytochrome (Zietz and Blomgren, 2006; Zietz and Gillbro, 2007). Studies on single dipyrrinone derivatives have yielded similar results, with rapid deactivation attributed to twisting motion of the two rings leading mainly to the recovery of the ground state *Z* isomer (Lamola et al., 1983; Zietz and Gillbro, 2007; Sakata et al., 2016). These results suggest the intrinsic nature of the dipyrrinone bilin subunit to undergo ring twisting in the excited state when free in solution, and a seminal work by Lightner *et. al.* elegantly demonstrated the suppression of this behavior by chemically linking the two rings. In that work, the fluorescence quantum yield went from Φ_F_ < 10^–4^ to 0.81 when the rings were constrained by a methylene bridge across the pyrrolic nitrogens (Hwang and Lightner, 1994). Recently, the same effect was demonstrated in a bilirubin fluorescent protein where Φ_F_ was found to be 0.51 due to the anchoring of the pyrrolinone ring by at least three direct hydrogen bonds from nearby amino-acids (Cao et al., 2019).

In this work, we seek to elucidate the local conformational landscape/flexibility, excited-state deactivation mechanism(s), as well as the spectroscopic properties of the two types of bilin dipyrrolic subunits absent of any bonds or interactions to an external scaffolding. Structures of the model dipyrrole core (DPY) and terminal dipyrrinone (DPN) subunits studied here are shown in Figure 1B. Steady-state spectroscopy and photophysical characterization was performed on each subunit, in conjunction with density functional theory (DFT) analysis of the conformational landscapes of each. In addition, femtosecond transient absorption spectroscopy was carried out to directly evaluate the excited state decay mechanism and the associated kinetics therein. Taken together, this bottom-up approach allows for a comprehensive analysis of the light capture ability and inherent excited-state torsional freedom of each part of the bilin framework, and those results precipitate the importance of specific interactions within the *in vivo* bilin environment in steering biological function.

## Materials and Methods

### Experimental

DPY **(**3, 5, 3′, 5′-Tetramethyl-1H, 2′H-2, 2′-methanylylidene-bis-pyrrole hydrochloride) was purchased from Sigma-Aldrich and used without further purification. DPN and N-methyl-DPN syntheses were adapted from Chepelev, et al. and Huggins et al. (Chepelev et al., 2006; Huggins et al., 2007). The reduced alkylation at the pyrrole site of the DPN derivatives studied here has been shown to significantly reduce facile dimer formation in nonpolar solvents; however, the dimer association constant, *K*_a_, for DPN in nonpolar solvents such as chloroform still reaches ∼3,850 M^-1^ due to extensive H-bonding at the pyrrolic amine and pyrrolinone carbonyl sites (Huggins et al., 2007). In order to disentangle monomer and dimer dynamics in solution, we compared the results of DPN with *N*-methyl-DPN (Figure 1B) which significantly inhibits H-bond formation that leads to dimer formation.

Steady-state UV-vis absorption spectroscopy was carried out on an Agilent 8453 UV-vis spectrometer, and molar extinction coefficients for each sample-solvent combination was determined by linear fitting of 4–5 different concentrations in the 10^–6^−10^–5^ M concentration range (A < 0.9) in a 1 cm quartz cuvette. The oscillator strength, *f*, for the S_0_−S_1_ transitions and the radiative rate constant, *k*_r_^0^, for each was estimated by calculation directly from the extinction spectra according to classical light theory (Turro et al., 2010). Corrected fluorescence spectra were collected using a PTI (Photon Technology International) fluorometer, and samples were contained in a 1 cm quartz cuvette with a maximum sample optical density (OD) of <0.3 to avoid inner filter effects. Fluorescence quantum yields, Φ_F_, were determined against references rhodamine 6G in ethanol solvent (Φ_F_ = 0.94) for DPY (Fischer and Georges, 1996), and 9,10-diphenylanthracene in ethanol (Φ_F_ = 0.88) (Mardelli and Olmsted, 1977) for DPN derivatives. Spectroscopic/photophysical properties for each sample were determined in both polar-protic methanol solvent and dichloromethane solvent for comparison. For DPY in methanol, 0.1 M HCl was added at 0.5% by volume to ensure complete protonation at both pyrrole sites in accordance with its native protonation state *in vivo* (Kneip et al., 1999; Rohmer et al., 2006; Corbella et al., 2018; Toa et al., 2019).

Transient absorption (TA) spectroscopy was undertaken at the Center for Ultrafast Optics and Lasers (CUOL) at Ft. Lewis College, and employed a 1 kHz Spectra-Physics Solstice Ace regenerative amplifier pumping a TOPAS Prime OPA for the pump pulse, while also seeding the white light generation stage of the Newport Transient Absorption Spectrometer. The white light continuum probe pulse was generated using a rotating CaF_2_ substrate. All TA measurements reported here were taken with the pump wavelength set to the peak of the S_0_−S_1_ transition for each sample, and the power was kept below 1 mW. The pump-probe polarization angle was set to magic angle, and each data set shown is an average of 10–20 scans each taken at 200 ms integration time. For TA experiments, samples were placed in a 1 mm cuvette with an OD < 0.5 at the peak absorption. Global analysis of the chirp-corrected data was performed using the Glotaran program (Snellenburg et al., 2012).

### Computational

All calculations were carried out in the Gaussian 16 suite (Frisch et al., 2016). Ground state geometry optimizations, harmonic frequency calculations, thermochemical calculations, and ground state potential energy scans were done using density functional theory at the M05-2X(Zhao and Truhlar, 2007)/6–311++G(d,p) level of theory with implicit solvation by methanol approximated using the polarizable continuum model (PCM). Time-dependent density function theory (TDDFT) at the same level of theory was used for vertical excitation calculations. Recent computational work on DPN using the hybrid semiempirical DFT/MRCI approach found that the first electronic transition of DPN resulted in negligible double-excitation character, justifying the TDDFT approach used here (Lyskov et al., 2020).

## Results

### Conformational Landscapes

**FIGURE 2:**
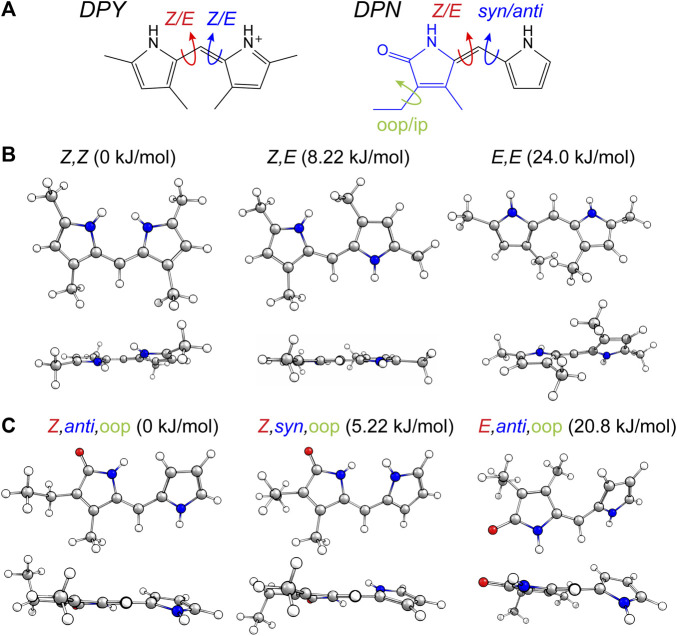
**(A)** Conformational degrees of freedom for DPY and DPN. **(B,C)** Calculated conformers of **(B)** DPY and **(C)** DPN with associated relative Gibbs free energies.

The torsional freedom of linear tetrapyrroles provides both spectral tunability when external contacts are made to contort the local geometry across a dipyrrole pair (such as the case in photosynthetic light harvesting), or the ability to isomerize across the methine bridge of a dipyrrole pair (photoswitching). The latter has been found to generally occur at the bilin terminus, however, this is largely due to covalent and electrostatic confinement of the core dipyrrole pair in the *in vivo* setting. In an effort to assess the local flexibility of each dipyrrole subunit isolated from these interactions, the ground state conformational minima of DPY, DPN, and *N*-Me-DPN were calculated along with the relative Gibbs free energies of each. Figure 2A shows the dominant nuclear degrees of freedom that separate the various conformational families found for DPY and DPN derivatives.

**FIGURE 3:**
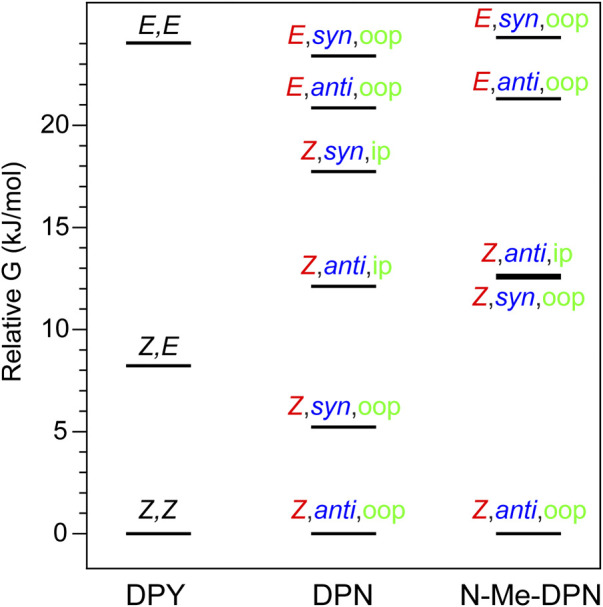
Gibbs free energy diagram for DPY, DPN, and *N*-Me-DPN conformational families.

For DPY, two-fold symmetry exists about the methine carbon given the protonated form studied here, leading to only three distinct conformers shown in Figure 2B. The DPY conformers differ in their torsional state across both methine bonds (C_4_–C_5_ and C_5_–C_6_). Given the resonance/symmetry in this case, the two torsional coordinates are identical and labeled as *Z* or *E* for each. The global Gibbs energy minimum structure is the *Z,Z* isomer with both NH groups facing one another, ultimately leading to a small N-C_4_--C_6_-N ring twist angle of ∼18°, while simultaneously minimizing the steric contributions of the methyl substituents. The same effect would be exacerbated in the fully alkylated case as is found *in vivo* to stabilize the *Z,Z* form. The other conformational minima are separated by significant Gibbs energy of 8.22 and 24 kJ/mol for the *Z*,*E* and *E*,*E* isomers. For the *Z*,*E* isomer, the first pyrrolic NH lies in the ring plane with the adjacent methyl group staggered about it to stabilize a fully planar structure. The *E*,*E* isomer structure reveals a highly twisted configuration (53°) motivated by the close proximity of flanking methyl substituents. This interaction pushes the conformational free energy significantly higher than the other *Z* forms. The relative Gibbs free energies are shown in Figure 3 for comparison. The additional steric interactions of the *Z*,*E* and *E*,*E* forms give way to Gibbs energies well above *kT*; therefore we expect only a negligible population at room temperature, likely not observable in experiment.

The ground state potential energy surface of DPN is significantly more complex than DPY due to the asymmetric nature of the pyrrolinone-pyrrole pair, along with the ethyl torsional freedom which adds to the heterogeneity of the ground state. Figure 2A gives the primary degrees of freedom for DPN along with the labels used to classify each. These include the twist coordinate about the methine double bond (C_4_–C_5_) separating *Z*,*E* families, the twist coordinate about the methine single bond (C_5_–C_6_) separating *syn*/*anti* configurations, and finally the pyrrolinone ethyl torsional configurations as either out-of-plane (oop) or in-plane (ip) relative to the pyrrolinone ring plane. The three lowest energy families are shown in Figure 2C, and already it is found by inspection that all conformers are nonplanar across the di-ring framework, generating degenerate pairs of minima associated with a positive or negative di-ring twist angle across the N-C_4_--C_6_-N dihedral. The ethyl group freedom further produces auxiliary minima associated with the oop configurations above or below the pyrrolinone plane. Therefore, there exists four near-degenerate minima for each “oop” family and two for “ip” families. For example, the *Z*,*anti*,oop conformational family contains four independent minima associated with combinations of +/− di-ring twist angles along with the two ethyl orientations. Nevertheless, the *Z*,*anti* torsional family is found to be the global minimum for both ethyl oop/ip, and is separated by ∼5 kJ/mol from the higher *Z*,*syn* family which positions the pyrrole NH groups adjacent one another. Interestingly, with a methyl substitution at the C_7_ position as in the DPY case, the ordering would likely be opposite. However, the nominal configuration of the torsionally-active pair in the phytochrome case is indeed the *Z*,*anti* conformation, so DPN is a representative model for that case. Comparing these families in the *N*-methyl substituted DPN (Figure 3), it is found that the *Z*,*syn* conformation is highly destabilized from the additional steric clash of the *N*-Me and adjacent amide NH.

### Spectroscopy and Photophysics

**FIGURE 4:**
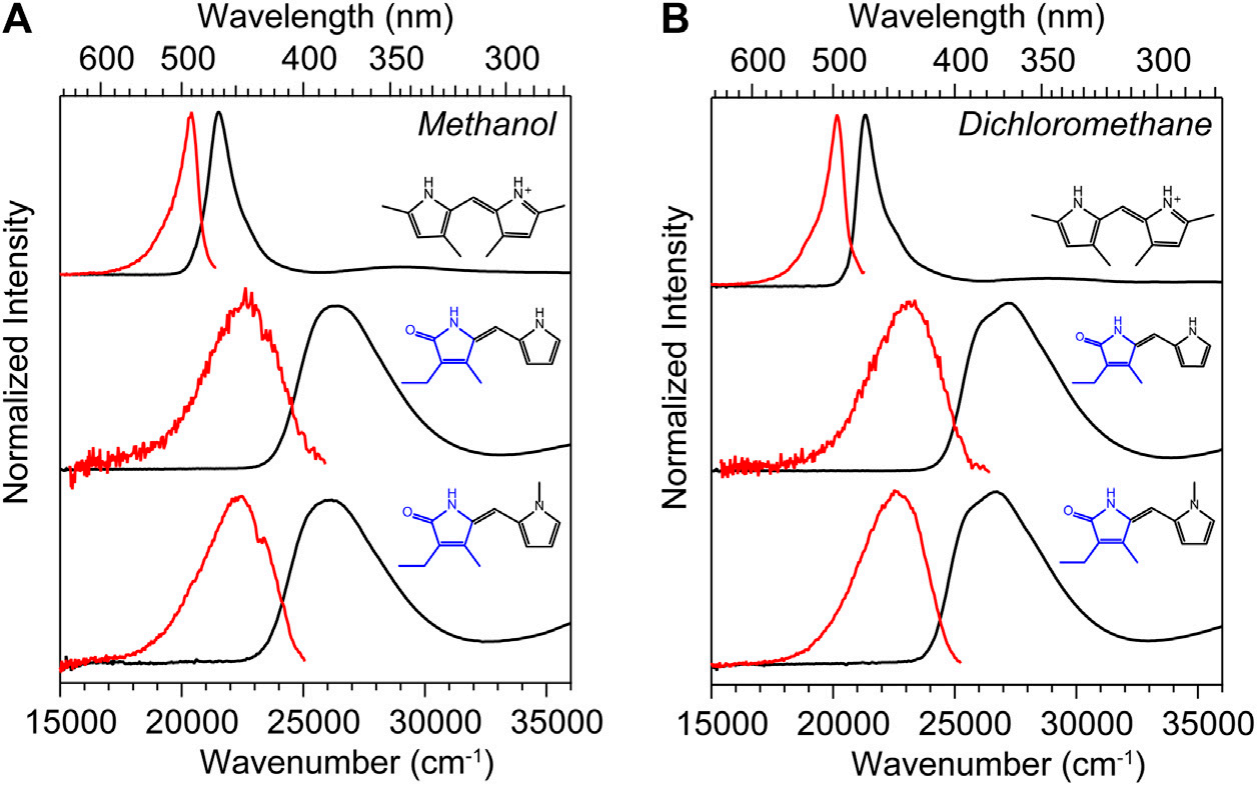
UV-vis absorption (black) and corrected fluorescence (red) spectra for DPY, DPN, and *N*-Me-DPN in **(A)** methanol and **(B)** dichloromethane.

The UV-vis absorption spectra (black) and corrected fluorescence spectra (red) are shown for all three compounds in methanol and dichloromethane solvents in Figures 4A,B respectively, and the wavenumber and wavelength positions for the absorption and emission maxima for each spectrum are given in Table 1 along with other observed and derived spectral properties. Immediately striking from a cursory comparison is the vastly different breadths of the absorption and fluorescence bands associated with the S_0_−S_1_ transition. DPY shows significantly more narrow bandshapes, and are red-shifted by nearly 5,000 cm^−1^ (85 nm) compared to DPN compounds leading to absorption well into the visible range (λ_max_ = 465 nm). We can attribute the broadening in DPN spectra at least in part to the conformational heterogeneity predicted by calculations, with the additional possibility of dimerization. However, calculation of the binding free energy for DPN and *N*-Me-DPN H-bonded dimers yields Δ*G*_dimerization_ = −2.77 and +12.1 kJ/mol respectively (structures given in Supplementary Figure S2 of the Supplementary Material). This suggests that dimerization of the *N*-methyl derivative should be minimal as anticipated. Despite this prediction, the breadth of spectra for both DPN compounds are very similar perhaps indicating a lack of substantial dimer formation at least in methanol. The small inflection at ∼23,000 cm^−1^ in the DPN and *N*-Me-DPN fluorescence spectra in methanol is due to incomplete subtraction of a methanol Raman band following subtraction of the methanol background.

**TABLE 1:** Spectroscopic properties of DPY, DPN, and *N*-Me-DPN in methanol and dichloromethane solvents determined from steady-state measurements. Band maxima are all given in wavenumbers (cm^−1^).

	υ^abs^_max_ (λ_max_, nm)	υ^fl^_max_ (λ_max_, nm)	Stokes Shift (cm^−1^)	*ε*_max_ (M^−1^cm^−1^)	*f*
DPY (MeOH)	21,505 (465)	20,408 (490)	1,097	102140	0.692
DPY (DCM)	21,322 (469)	20,161 (496)	1,161	147390	0.965
DPN (MeOH)	26,316 (380)	22,573 (443)	3,743	32,610	0.641
DPN (DCM)	27,174 (368)	23,148 (432)	4,026	26,480	0.51
N-Me-DPN (MeOH)	26,178 (382)	22,371 (447)	3,807	32,340	0.657
N-Me-DPN (DCM)	26,738 (374)	22,676 (441)	4,062	25,880	0.528

Reflecting on the global minimum conformers of DPY and DPN, the red-shift associated with DPY is at least partially associated with a smaller inter-ring twist angle (18° vs. 30°)―a property of bilins which has been shown to drastically tune the absorption wavelength (Tang et al., 2015; Wiebeler et al., 2019; Xu et al., 2020). However we cannot rule out the possibility of the influence of the DPN carbonyl on the electronic structure itself. According to vertical excitation energy calculations however, the S_0_−S_1_ transitions are described exclusively by a HOMO→LUMO (π→π*) excitation for both derivatives showing nominally the same electron delocalization length. Another remarkable feature of all spectra in Figure 4 is the dramatic Stokes shift between band maxima of ∼1,000 cm^−1^ (∼25 nm) for DPY and ∼4,000 cm^−1^ (∼63 nm) for DPN. Given the similarity in lineshapes, the massive wavenumber difference must be due in part to a dramatic geometry change between S_0_ and S_1_, and in the case of DPN possibly an emissive conformer population among many separately absorbing populations.

Table 1 summarizes the absorption strength of each molecule via their molar extinction coefficients (at λ_max_) along with the calculated oscillator strengths for the electronic transition. All compounds demonstrate large oscillator strengths for their S_0_−S_1_ transition, on average *f* ∼ 0.6. This is a remarkable result given their relatively small and atomically “cheap” structures, and highlights the effectiveness of this naturally-designed molecular framework for light capture. We note that despite the smaller *ε*_max_ measured for DPN compounds, the integrated intensity of the broad absorption yields an oscillator strength comparable to its DPY counterpart. As predicted, the absorption wavelength and band shape of DPY is found to be very similar to the phycourobilin chromophore found in cyanobacterial phycobiliproteins, and as a result the peak molar extinction coefficient is also very comparable (*ε*_max PUB_ = 104000 M^−1^ cm^−1^) (Glazer and Hixon, 1977). Notably, various forms of chlorophyll yield a *ε*_max_ of 35,000–90,000 M^−1^ cm^−1^ for the red-most *Q*_y_ transition with comparable widths to DPY, suggesting the chemically simpler pigments studied here may be even better light absorbers in their respective spectral regions (Scheer, 1991).

Table 2 gives photophysical characteristics of each of the sample/solvent combinations. *k*_r_ is the radiative rate constant calculated from the integrated intensity of the main absorption band, and τ_r_ is therefore the natural lifetime and falls in the typical nanosecond time scale. The fluorescence quantum yields of all compounds are exceedingly low (∼10^–4^), in agreement with previous studies on dipyrrinones (Hwang and Lightner, 1994). We speculate then, that the torsional motion activated following electronic absorption is as extensive as other free dipyrrinones in solution. Taking these data together, the total nonradiative rate constant, *k*_nr_, for each and its associated time constant are also given in Table 2. One can see that deactivation of the S_1_ excited molecule takes place on average in a few picoseconds or less. DPY in methanol yields the smallest Φ_F_ and therefore fastest nonradiative decay at ∼500 fs.

**TABLE 2:** Photophysical properties of DPY, DPN, and *N*-Me-DPN in methanol and dichloromethane solvents taken from steady-state measurements.

	k_r_ (s^−1^)	*τ*_r_ (s)	Φ_F_	*k*_nr_ (s^−1^)	*τ*_nr_ (s)
DPY(MeOH)	2.23E+08	4.48E-09	1.15E-04	1.94E+12	5.15E-13
DPY (DCM)	3.06E+08	3.27E-09	6.02E-04	5.08E+11	1.97E-12
DPN (MeOH)	3.13E+08	3.19E-09	3.43E-04	9.13E+11	1.10E-12
DPN (DCM)	2.63E+08	3.80E-09	1.01E-03	2.60E+11	3.84E-12
N-Me-DPN (MeOH)	3.14E+08	3.18E-09	2.17E-04	1.45E+12	6.91E-13
N-Me-DPN (DCM)	2.63E+08	3.80E-09	6.16E-04	4.27E+11	2.34E-12

### Transient Absorption Spectroscopy

**FIGURE 5:**
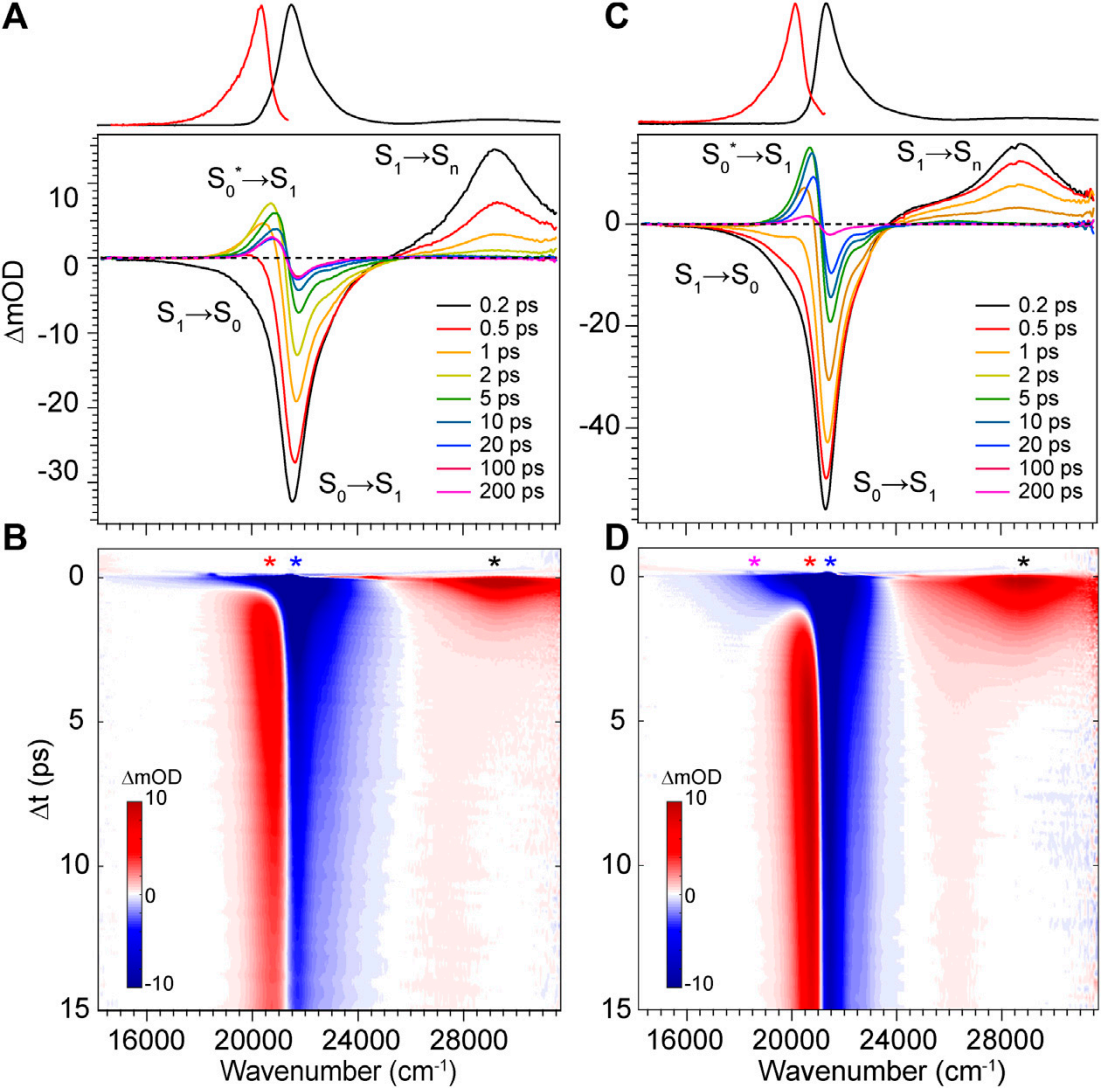
Transient absorption spectra for DPY in **(A,B)** methanol and **(C,D)** dichloromethane. Asterisks in b and d denote wavenumber positions for transients shown in Figure 7. Absorption (black) and fluorescence (red) spectra are shown at the top for reference.

To elucidate the exact mechanism for rapid excited state decay of DPY and DPN, transient absorption spectroscopy was undertaken for each. Figures 5A,B show the evolution of transient absorption spectra of DPY in methanol, with the absorption and fluorescence spectra given above as reference. At very early times, the symmetrical feature incorporating both negative-going S_0_−S_1_ ground state bleach (GSB) and S_1_−S_0_ stimulated emission (SE) signals can be immediately identified, and is centered at 21,500 cm^−1^. In addition, a large excited state absorption (ESA) signal is present at 29,250 cm^−1^ at time zero. Before a dynamic Stokes shift can be observed however, the SE signal disappears within ∼500 fs as a new photo-induced absorption (PIA) signal around 20,400 cm^−1^ appears within 1 ps. Within the same timeframe, the ESA signal to the blue decays and significant GSB is lost. Within 15 ps, nearly all of the ESA signal has vanished while most of the ground state has been re-populated―signaling facile internal conversion. During this time, the induced absorption signal peaks around 2.5 ps and continuously narrows and blue-shifts before itself decaying away to reveal a broader PIA signal after 10 ps. This early time behavior is indicative of formation of a hot ground state population, S_0_^*^, immediately after the internal conversion curve-crossing event at the S_1_ potential energy minimum. As the population cools, the PIA signal blue-shifts and narrows before disappearing upon reaching equilibrium in the ground state. While similar ESA and SE behavior could be expected with a substantial geometry change as the molecule leaves the Franck-Condon region of the S_1_ surface, the concomitant loss of GSB can only be explained by cooling within the ground state. Interestingly though, a fraction of the GSB signal remains even at 3 ns along with the broader positive signal at 20,800 cm^−1^.

The dynamics in dichloromethane solvent (Figures 5C,D) are nearly identical except for occurring at a lower rate. The slower evolution in these data clearly bear out the cooling behavior of the hot ground state population and finally the remaining product population at lower yield. The slower deactivation of the S_1_ state in dichloromethane is somewhat unexpected given the viscosity and polarity are both reduced compared to methanol (thereby enabling less hindered twisting about the methine bridge). Therefore, we surmise that H-bonding interactions between DPY and the polar protic methanol solvent must manipulate the excited potential energy surface to allow the excited molecule to sample the conical intersection more efficiently. Alternatively, the H-bonding at the pyrrole amines may alter the electronic structure directly leading to a reduced bond order of the C-C bonds making up the methine bridge.

**FIGURE 6:**
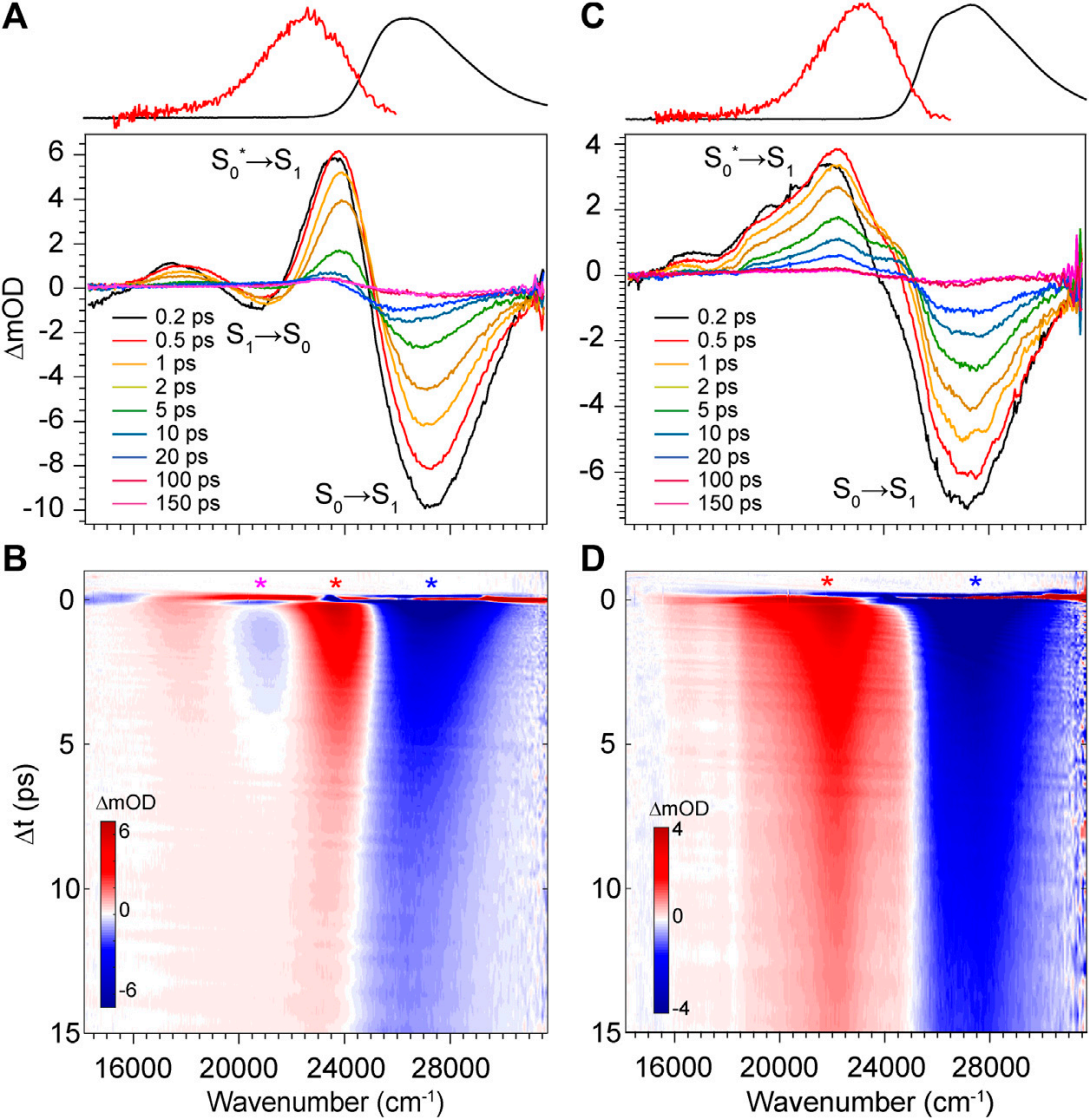
Transient absorption spectra of DPN in **(A,B)** methanol and **(C,D)** dichloromethane. Asterisks in b and d denote wavenumber positions for transients shown in Figure 9. Absorption (black) and fluorescence (red) spectra are shown at the top for reference.

The transient absorption spectra and time evolution for DPN in methanol and dichloromethane are given in Figure 6. Immediately after excitation, the GSB peaks just blue of the absorption band due to a positive PIA peaking at 23,580 cm^−1^ present already within the time resolution of the experiment. The signal spans out as far as 16,000 cm^−1^, with a negative SE signal appearing at ∼21,000 cm^−1^ separating the two sections of the broad positive feature. Similar to the results of DPY, the ground state rapidly refills (loss of GSB) coincidentally with the blue-shift and narrowing of the PIA. This behavior again signifies cooling on the ground state surface following an internal conversion event. Internal conversion in this case must occur within the pulse overlap time (<120 fs) given the immediate appearance of the hot ground state signature. Curiously though, the residual SE signal decays in this case within ∼5 ps―much longer than the proposed internal conversion timescale. To this point, it is found that between 5 and 20 ps, as the hot ground state population has thermalized, a second population is revealed and evolves with slower kinetics. This is observed in the remaining GSB signal at ∼20 ps which is red-shifted by 1,000 cm^−1^ from the early time GSB. A marked feature of these distinct species is the seemingly red-shifting inflection between the primary GSB and PIA signals in Figure 6B. Finally, this signal decays over the next 50 ps to lastly show a very small and broad GSB around 27,000 cm^−1^ with residual PIA ∼23,000 cm^−1^. Similar spectral dynamics are observed in this progression (shifting GSB/PIA inflection) except toward the blue direction; these longer time data can be found in Supplementary Figure S3 of the Supplementary Material. The sum of these observations points to at least three independent ground state populations. These species could be various DPN conformers given the increased complexity of the DPN potential energy surface (Figure 3), and/or some dimer contribution in addition.

To address this question, we performed TA spectroscopy on the *N*-methyl DPN derivative which locks out dimer formation by eliminating two H-bond sites. These results are given in Supplementary Figure S4 of the Supplementary Material, and show nearly identical spectral features with remarkably similar dynamics, except for the final remaining GSB/PIA signals which are absent in those data. In fact, unlike DPN, all of the population has returned entirely to the ground state by the end of the 200 ps experiment. The longer-lived blue-most absorbing population in DPN can therefore be tentatively assigned to a small fraction of dimer in solution, with the intermediate red-absorbing population assigned to the *Z*,*syn*,oop family of conformers. Boltzmann analysis yields ∼12% of the total population belonging to this family at thermal equilibrium, and is consistent with the relative GSB intensities shown in Figure 6.

The TA data for DPN in dichloromethane (Figures 6C,D) shows two primary signals peaking ∼27,350 cm^−1^ and 22,150 cm^−1^. Unlike in methanol, these features decay directly with approximately the same kinetics and little to no spectral changes therein. In this case, the positive signal at 22,150 cm^−1^ is then assigned to an ESA given the static band profile. Keeping in mind the large dimer association constant of DPN in chloroform, this single species is assumed to be (DPN)_2_, and its excited-state dynamics are dominated by internal conversion with a time constant of ∼3 ps.

## Discussion

### Excited State Decay Mechanism of Model Dipyrrole Subunits

**FIGURE 7:**
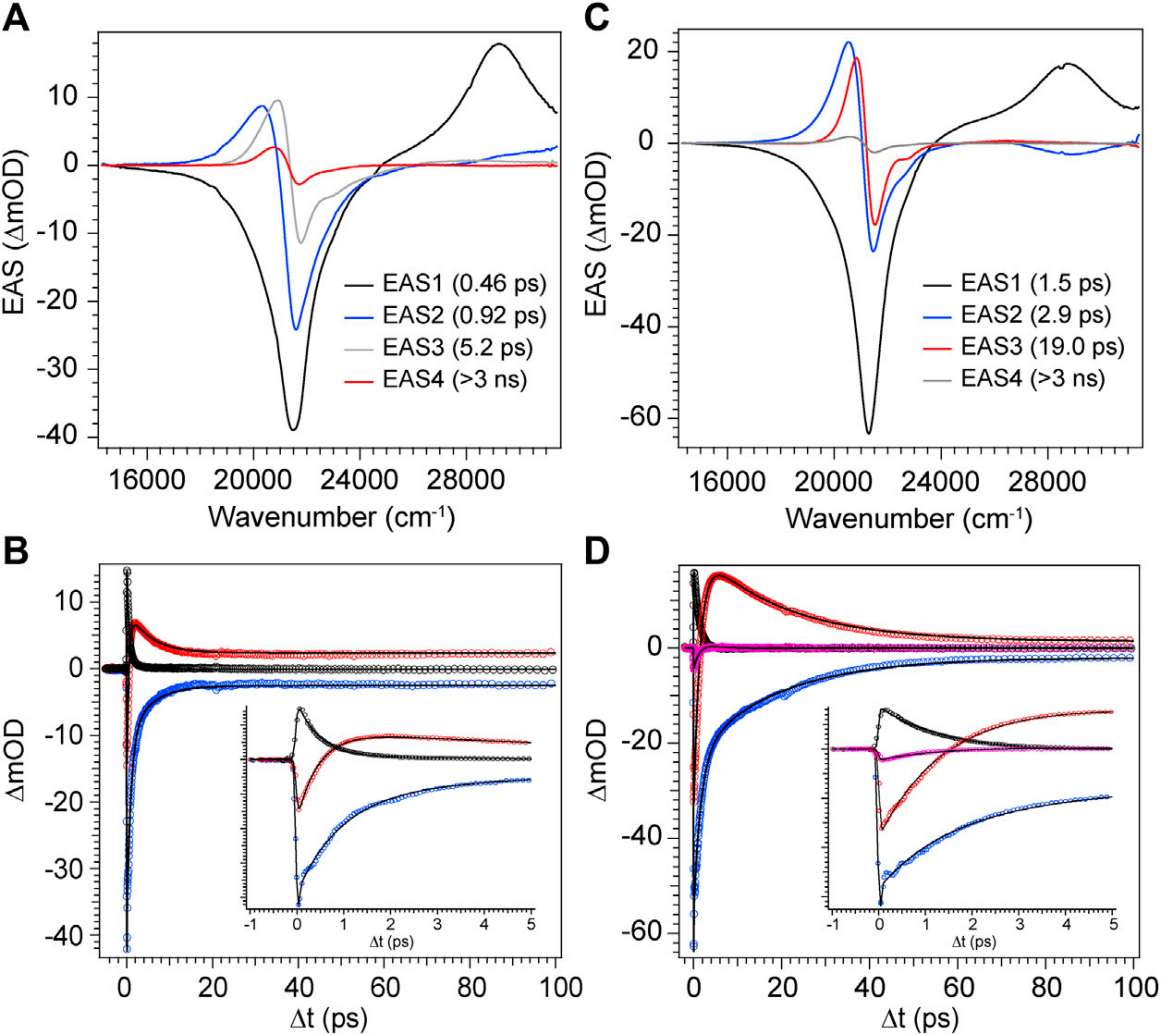
Global analysis results from DPY TA data. **(A,C)** Evolutionary-associated spectra (EAS) with associated time constants for DPY in **(A)** methanol and **(C)** dichloromethane. **(B,D)** Selected experimental transients (open circles) with fits from the employed global sequential model for DPY in **(B)** methanol and **(D)** dichloromethane.

With the combination of photophysical data derived from steady-state spectroscopy measurements and time-resolved transient absorption, we are now in a position to address the excited state behavior of these model bilin subunits free in solution. First, in order to determine the exact time/rate constants associated with the excited state decay pathways of DPY and DPN, global analysis of the chirp-corrected data was performed. For DPY, a sequential model was applied to the data given the homogeneity of the ground state population. The evolutionary-associated spectra (EAS) along with the fit to selected transients (positions marked with asterisks in Figure 5) are given in Figures 7A,B respectively for DPY in methanol.

A total of four components was required to capture the somewhat complex evolution of all features. The component represented by EAS1 decays with τ_1_ = 0.46 ps and clearly captures the SE, initial GSB, and ESA band near 29,000 cm^−1^. We therefore assign this component to direct internal conversion and highlight that the timescale is on the order of torsional nuclear motion. EAS2 shows a significantly reduced GSB signal along with the PIA peaking near 20,300 cm^−1^ and trailing to the red. The decay of these features at τ_2_ = 0.92 ps to EAS3 with the PIA now blue-shifted by 600 cm^−1^ indicates the first mode of vibrational cooling of the hot ground state population, S_0_^*^. Finally EAS3 decays on a 5.2 ps timescale associated predominantly with a second mode of vibrational relaxation given the still large reduction in GSB and PIA from EAS3→EAS4, and is likely from a bottleneck near the ground state minimum where the state density drastically decreases (Owrutsky et al., 1994). The final EAS4 is associated with the remaining signals at the end of the experiment and apparently static as found in Figure 7B at longer times. This remaining population is assigned to the fraction of molecules that underwent *Z*→*E* isomerization to generate a new ground state photoproduct, and we surmise that EAS3 also incorporates this small fraction of isomerizing population occurring on a similar timescale since no other components were needed to fit the data.

The global analysis results for dichloromethane experiments are shown in Figures 7C,D and are qualitatively similar to the results in methanol. The features are sharper due to the reduced inhomogeneous broadening of the nonpolar solvent, and as anticipated the time constants for each mode of decay are larger. This is somewhat surprising in the initial internal conversion step as indicated previously, but the subsequent vibrational cooling steps are expectedly longer due to significantly weaker ion-dipole and H-bonding interactions with the protonated DPY (Owrutsky et al., 1994). A summary of all time constants from global analysis can be found in Table 3.

**TABLE 3:** Time constants extracted from global analysis of transient absorption data for DPY, DPN, and *N*-Me-DPN.

	DPY (MeOH)	DPY (DCM)	DPN (MeOH)	DPN (DCM)	N-Me DPN (MeOH)
τ_1_ (ps)	0.463	1.47	2.51	2.89	2.17
τ_2_ (ps)	0.915	2.93	44.7	19.0	23.1
τ_3_ (ps)	5.19	19.4	>3000	>3000	-
τ_4_ (ps)	>3000	>3000	-	-	-

**FIGURE 8:**
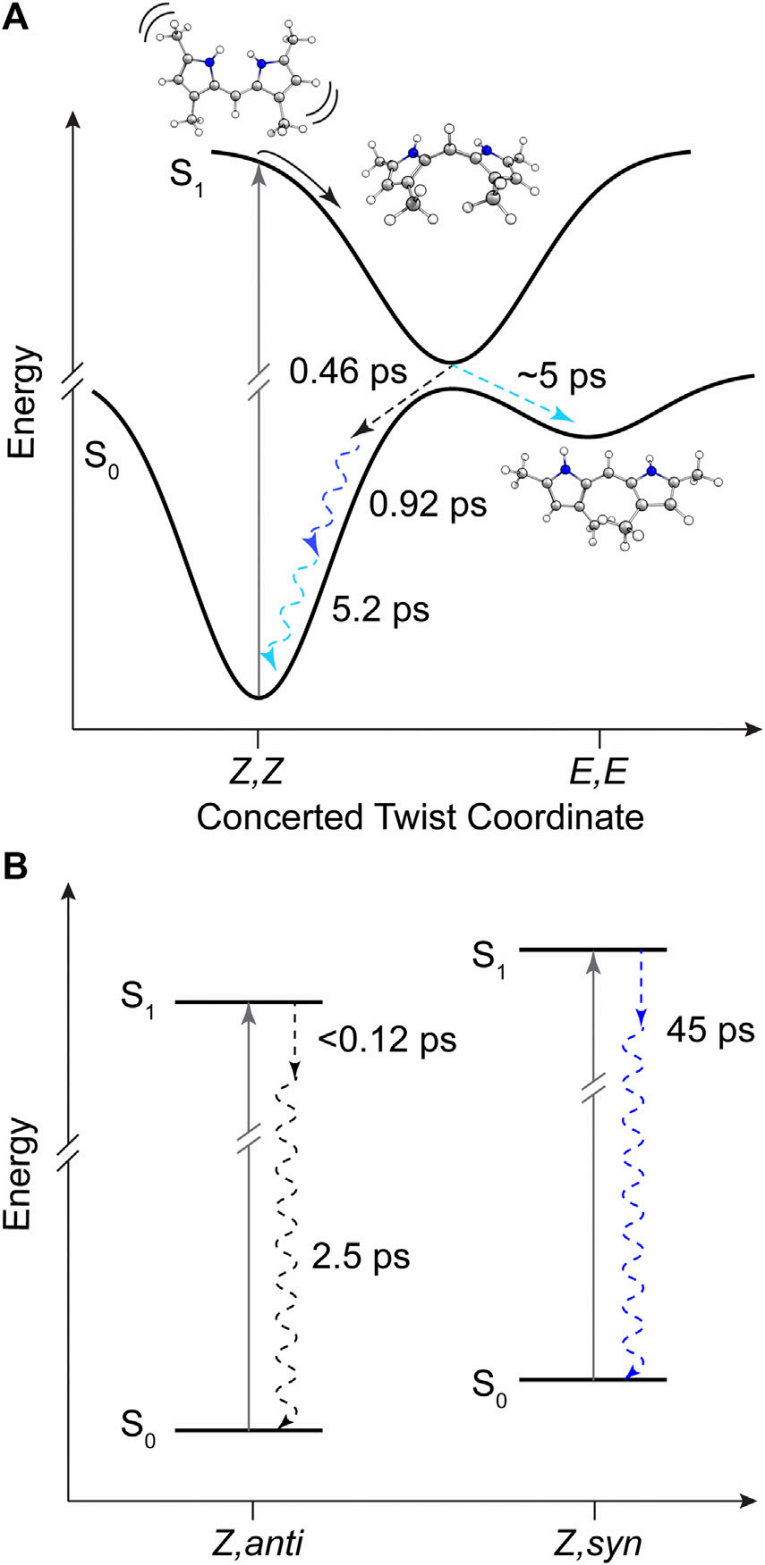
Summary of DPY and DPN excited state dynamics in methanol. **(A)** Representative potential energy curves of DPY showing rapid torsional relaxation of the excited population toward a conical intersection with the ground state. **(B)** Energy level diagram for DPN populations in solution with time constants for decay. Dashed arrows represent dynamics captured in global analysis and are color-coded according to Figures 7 and 9 respectively. Squiggly arrows indicate vibrational relaxation.

The product absorption of the photoisomer shown in EAS4 in both cases is red-shifted from the *Z*,*Z* isomer. Evaluating the TDDFT vertical excitation energies of all conformers (Supplementary Material), only the *E*,*E* isomer has an absorption red of the *Z*,*Z* global minimum. The photoproduct can then be tentatively assigned to the high energy *E*,*E* conformer, suggestive of a mechanism involving concerted twisting about both C_4_-C_5_ and C_5_-C_6_ bonds. Such a scenario was predicted for an unalkylated DPN derivative previously (Zietz and Blomgren, 2006), and can be rationalized in this case by the symmetric nature of the DPY molecule. The S_0_−S_1_ electronic transition is effectively a HOMO→LUMO transition according to TDDFT results, and inspection of those molecular orbitals (Supplementary Figure S5) reflect their symmetric nature (resonance) and consequently symmetric bond order change. Thus, the electronic transition serves to simultaneously elongate both methine bridge bonds in response to their increased antibonding character in S_1_. In support of this conclusion, when comparing the DPY conformers the inter-ring twist angle of the *E*,*E* configuration is found to be the most twisted by far (53°). Given the expected ∼90° twisted excited state minimum common to dipyrroles, the *E*,*E* ground state minimum would therefore be nearest to the conical intersection ultimately presenting the first "trap" to the isomerizing population. A representative potential energy diagram summarizing the excited state dynamics of DPY in methanol is given in Figure 8A.

**FIGURE 9:**
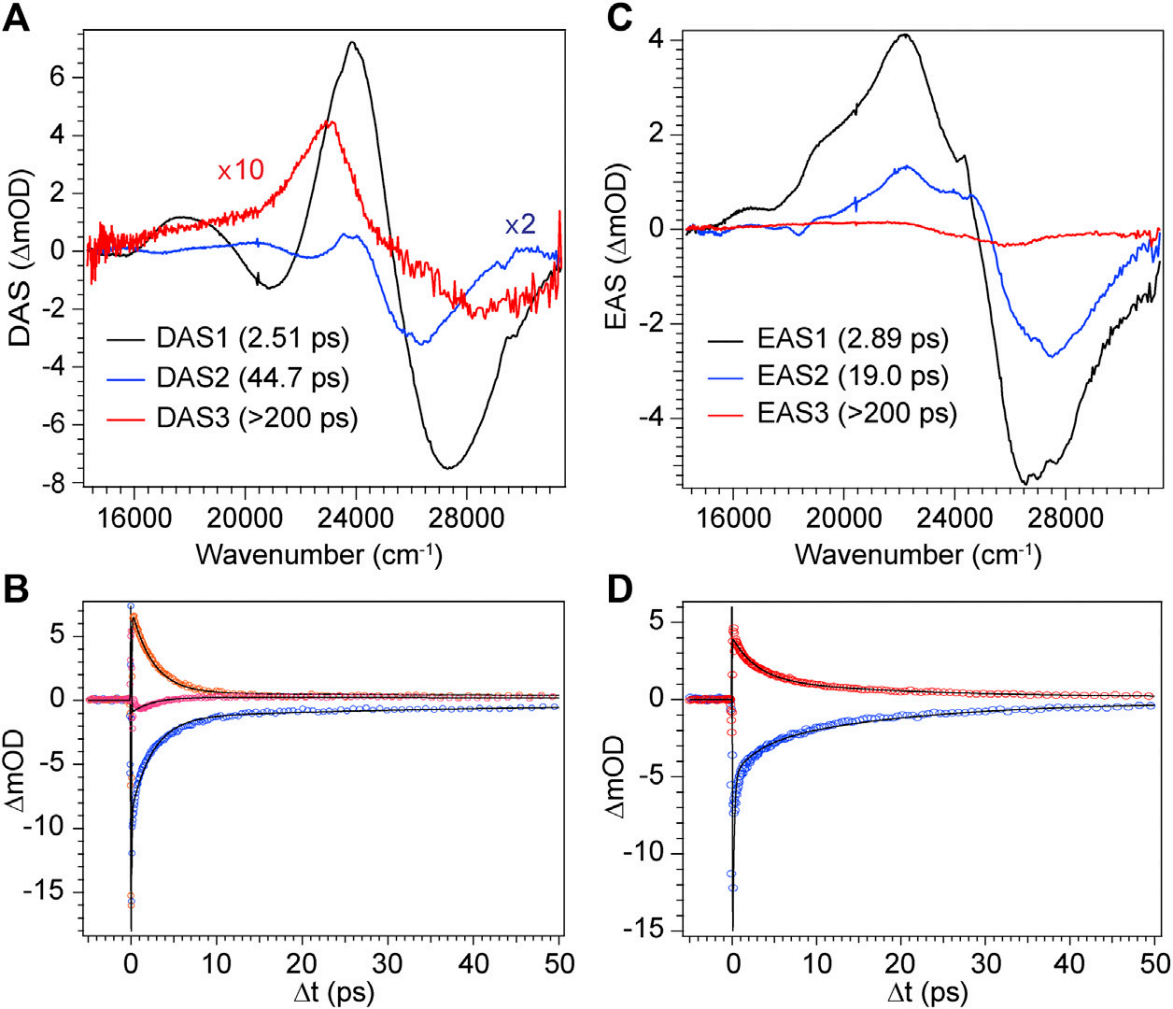
Global analysis results from DPN TA data. **(A)** Decay-associated spectra (DAS) with associated time constants for DPN in methanol and **(C)** EAS with associated time constants for DPN in dichloromethane. **(B,D)** Selected experimental transients (open circles) with fits from the employed global model for DPN in **(B)** methanol and **(D)** dichloromethane.

The decay of excited DPN in methanol was modeled with independently decaying components, and the decay-associated spectra (DAS) are shown in Figure 9A. As mentioned previously, the appearance of the hot ground state population (via the band-shape and spectral evolution of the PIA signal red of the GSB) immediately after the coherent artifact suggests that the internal conversion event for the dominant DPN population (*Z*,*anti*,oop monomer) occurs within 120 fs. This assignment can be substantiated by the comparable spectral dynamics of the PIA near 24,000 cm^−1^ to that of DPY. The first resolvable component, DAS1, then is associated with the primary mode of vibrational cooling of that internally converted population within the ground state. DAS2 decays with a 44.7 ps time constant and captures the red-shifted GSB near 26,000 cm^−1^. This component can be assigned to the minor *Z*,*syn* conformer population, and the DAS partially incorporates a SE signal peaking very close to the steady-state fluorescence spectrum. We can therefore assume that this longer-lived population is the primary emitting species, and the red-shifted absorption compared to *Z*,*anti* then partially explains the extensive Stokes shift in Figure 4A. DAS3 represents the signals remaining at the end of the 200 ps experiment and is assigned either to a residual dimer population based on its absence in the *N*-Me-DPN data, or to a very small population of isomerized product which is also absent in *N*-Me-DPN. The latter can be justified considering the added steric contribution of the bulky amino methyl group to inhibit the isomerization reaction. The agreement between experiment and model was quite good, as demonstrated by the fit to selected transients in Figure 9B. A representative energy level diagram summarizing these dynamics is given in Figure 8B.

DPN in dichloromethane solvent is presumed to be dominated by H-bonded dimers, and the two primary TA signals decayed with very little change in band structure. As such, the data was successfully fit to a sequential model and the results are given in Figures 9C,D. Comparison of EAS1 and EAS2 show that about half of the excited population has returned to the ground state within 2.89 ps. EAS2 is very similar to EAS1 and is either a separate dimer population or vibrational relaxation within the ground state, or a combination of both. At the end of the experiment, EAS3 shows some remaining population which has not yet returned to the ground state, with an intensity similar to DAS3 in Figure 9A. We note that in both solvents, the GSB intensity immediately after time zero is drastically lower in magnitude compared to DPY despite similar initial steady-state absorbances. Such a result implies that much of the GSB is lost within the pulse overlap time for DPN in both solutions, including for dimers present in solution, consistent with dynamics dominated by ultrafast internal conversion.

The steady-state photophysics (Table 2) and TA analysis are in excellent agreement regarding the rate of deactivation of excited DPY when taking τ_1_ as τ_IC_. This yields a non-radiative rate constant, *k*_nr_, of 2.17 ps^−1^ (τ = 0.461 ps) and 0.67 ps^−1^ (τ = 1.49 ps) for methanol and dichloromethane solvents compared to 1.94 ps^−1^ (τ = 0.515 ps) and 0.51 ps^−1^ (τ = 1.96 ps) found from steady-state results. The heterogeneity of the DPN ground state precludes this comparison for DPN.

In all samples, the decay pathway dominating the rate was direct internal conversion to the original ground state in <3 ps. Furthermore, the ultrafast decay (<120 fs) of the dominant *Z*,*anti* DPN species highlights the enhanced flexibility of the terminal bilin subunit when in an analogous starting conformation to biological phytochromes, in agreement with photosensory biliproteins and conformational results. With this in mind, *Z*→*E* isomerization was only definitively observed for DPY, at least in part due to the lower internal conversion rate. Interestingly, the *E*,*E* conformer was formed in the process as the ground state configuration lies closer to the excited state relaxed geometry. These results implicate a symmetric, concerted twisting mechanism for DPY photo-induced isomerization.

### Nature’s Selection of Multi-Pyrrolic Pigments and Design Motifs for Bilin Function

The remarkably large oscillator strengths found for these chemically simple dipyrroles immediately illustrates Nature’s “selection” of the pyrrole class of compounds as the base unit for light capturing pigments in nearly all biological contexts. In addition, comparing the absorption regions for the seemingly similar DPY and DPN frameworks reveals an extraordinarily large difference of nearly 100 nm with the former absorbing blue light and the latter near-UV. This “design” characteristic/sensitivity provides a synthetically efficient method to tune the region of light capture for photosynthetic organisms depending on available light quality, and further corroborates the functional connection between bilin derivatization *in vivo* and spectral tuning.

Outside of these ideal static spectral properties, the dynamics following photon absorption are of utmost importance to the final function of the natural biliproteins which bind the bilin. The dynamics observed in these simpler, free subunits reiterates the inherent tendency for large amplitude torsional motion within any multi-pyrrolic system following an electronic transition. While the terminal subunit indeed has a greater propensity for this behavior, as predicted from natural bilin photosensors, both regions of the bilin framework are prone to large-scale inter-ring twisting. Free of a protein scaffolding to bind or facilitate a controlled twist, this free motion deactivates the excited molecule rapidly back to its ground state before any sizable *E*-isomer population is generated. To avoid this loss in the phytochrome case (*Dr*BphP), the A−C pyrrole rings all contain at least one anchor to the protein scaffold with ring A being covalently attached by a Cys linkage, and rings B and C anchored by propionate groups at the C_3_/C_7_ positions which electrostatically attach to nearby Arg, His, and Ser amino acids (Rockwell et al., 2006). Additionally, the positive charge of the protonated core delocalized over rings B and C is closely associated with the negative charge and C=O of local His and Asp residues. These structural features ultimately restricts any twisting of the core and locks the bilin center of mass to the protein scaffold. The isomerizing ring D however, is contorted out of the bilin plane via electrostatic interaction with a nearby histidine (His290), but is generally less confined than rings A−C. Other than the single H-bond to His290, the binding pocket surrounding ring D is hydrophobic with phenyl rings from neighboring Phe residues “boxing” off the sides of the pocket (∼4 Å distance) with Met groups capping the top. In the multistep isomerization process, this binding pocket plays a vital role in stabilizing the Lumi-R intermediate and P_fr_ product, and the individual steps of the mechanism are highly coupled events between bilin and protein (Rockwell et al., 2006; Ulijasz and Vierstra, 2011). Similarly, in the prominent red/green family of cyanobacteriochromes, the indole group of a nearby Trp residue closes the binding pocket around ring D via a π-stacking interaction (3.5 Å) in the native/dark state, which is then displaced following photon absorption by some 14 Å allowing an influx of water into the binding pocket (Xu et al., 2020). Both scenarios reveal a local malleability at least near the ring D region of the chromophore to confer the concerted structural changes needed to stabilize the photoproduct.

To compare the phytochrome scenario to the functionally-opposing case of photosynthetic light-harvesting, it is instructive to assess the local pigment-protein binding motifs of the various types of bilins associated with a model phycobiliprotein antenna complex. To that end, we have chosen the highly studied PC645 protein from *Chroomonas sp.* which incorporates two nearly identical sets of phycocyanobilins (PCBs), mesobiliverdins (MBVs), and dihydrobiliverdins (DBVs) for the two near-symmetric αβ halves. We note that amino acid sequences of this class of phycobiliproteins are highly conserved (Harrop et al., 2014), and comparison herein is considered general for cryptophyte phycobiliproteins.

Firstly, all of the bilins in PC645 are covalently attached to the protein at the ring A position by a Cys linkage, and the DBVs in the center of the protein have an additional linkage at ring D to immediately anchor that part of the otherwise torsionally-active part of the chromophore. This is especially crucial in the DBV case to inhibit twisting, as it is the only bilin to incorporate a methylene bridge (C-C-C) instead of a methine bridge between rings C and D naturally increasing the flexibility at its terminus. The methylene bridge however blocks electronic conjugation out to ring D enabling DBVs to capture light further blue compared to the other incorporated bilins. The additional Cys linkage at ring D is therefore a targeted design motif to restrict excitation losses due to twisting about the methylene, while still expanding the spectral cross section of the protein to the blue region.

**FIGURE 10:**
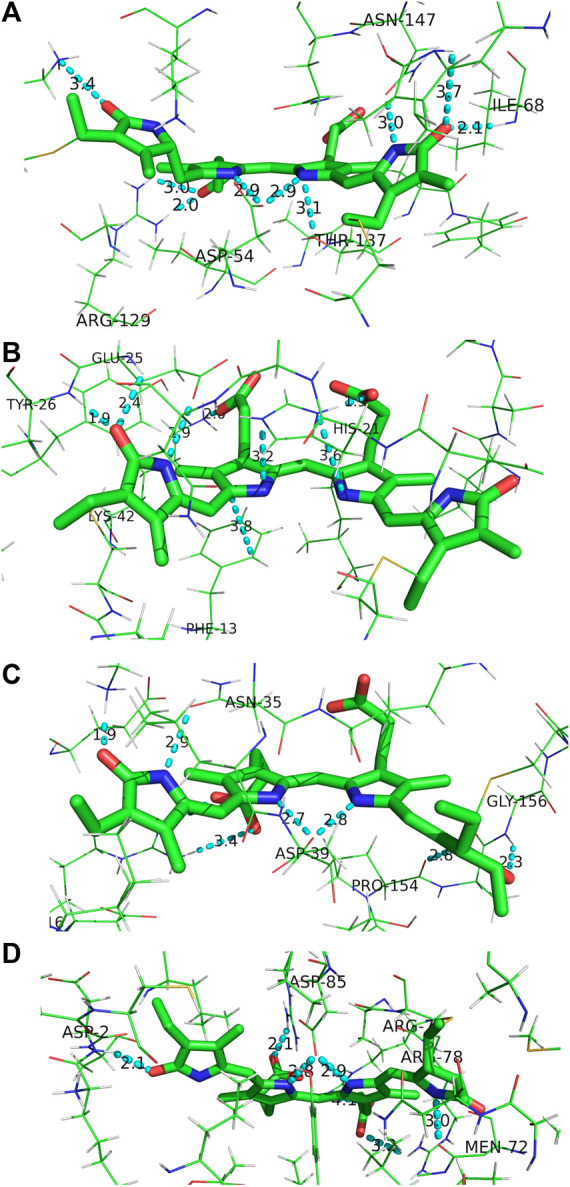
Binding pocket surrounding **(A)** DBV, **(B)** MBV, **(C)** PCB158, and **(D)** PCB82 found in the PC645 crystal structure.

The local binding pocket of the DBV, MBV, PCB158, and PCB82 bilins are shown in Figure 10. Beginning with DBV (β-subunit D, Figure 10A), ring A (right) is found to participate in three H-bonds with Ile68 and Asn147 in addition to the Cys covalent bond, and also stacking within 3.8 Å of the neighboring DBV. The core dipyrrole is secured on both sides, with the propionic tail of ring C within 2.0 Å of the guanidine group on Arg129, and the Asp54 carboxylate straddling the central rings within 3 Å. The latter feature has also been shown to stabilize the protonated form of DBV and PCBs under physiological conditions (Corbella et al., 2018). Lastly, the ring D carbonyl is in line with the Lys67 NH_3_^+^ group to further lock the ring in place at a position adjacent to its Cys linkage. We note that all of the bilins nominally have propionic groups pointing outward to the protein-solvent interface, and MBVs and PCBs are relatively close to the interface compared to the imbedded DBVs. Seemingly in all biliproteins, the propionic tails of the B and C rings help lock in the planarity of the bilin core to restrict excessive twisting of the center of the chromophore. Comparing with the torsional freedom of DPY which lacks these auxiliary groups to the electronic system, this feature is an obvious design requirement *in vivo* to ensure no twisting will occur at the center of the chromophore. In addition, the ubiquitous planarity of the core dipyrrole ensures a conjugation length that spans at least two pyrrole units resulting in an absorption always in the visible region, as demonstrated by the absorption spectrum of DPY.

For MBV (Figure 10B) covalently attached to the α subunit, ring A (right) is primarily anchored by the single Cys linkage, and rings B and C are again locked in place via interactions between propionic carboxylates with proximate (∼2 Å) His21 imidazole N and Lys42 ammonium groups. In addition, ring C is sandwiched by His21 and the phenyl ring from Phe13. Finally, ring D is held *anti* to ring C at a NC--CN twist angle of 113° by three H-bonds with the carbonyl acceptor engaged in a strong H-bond with the Tyr26 amide NH in addition to a 2.4 Å H-bond with the neighboring Glu25 residue. Finally, the Glu25 carboxylate is within 3.0 Å of the ring D NH group locking in the large twist angle. Presumably this latter feature is what blue-shifts the MBV absorption relative to PCB despite its more extensively conjugated chemical structure spanning all four rings (Tang et al., 2015; Wiebeler et al., 2019). This intermediate absorption gives way to enhanced capture between DBVs and PCBs and fills out the center of the otherwise bimodal absorption spectrum. This central MBV band can indeed be observed at 77 K (Dean et al., 2016).

Next, the PCB158 (β-subunit D, Figure 10C) is situated at the solvent interface of the β subunit with the propionic groups likely solvated. Ring A accepts a H-bond from the Gly156 amide, and donates one to a neighboring Pro amide carbonyl. Similar to the β-subunit bound DBV, rings B and C are straddled by a Asp carboxylate locking the two in a planar configuration. Ring D is engaged in a strong ion-dipole interaction between the pyrrolinone C=O and Lys28 ammonium end group less than 2 Å away. A secondary interaction to lock in the slightly contorted inter-ring conformation (133°) is from a H-bond of the pyrrolic NH to a Asn carbonyl.

Finally, the terminal energy acceptor, PCB82 (β-subunit D, Figure 10D), similarly situated at the periphery of the protein, takes on a more planarized local conformation with the NC--CN dihedral angle between rings D and C at 168°. This binding pocket-steered conformation ensures that PCB82’s absorption is the most red-shifted among the bilin network to place it at the bottom of the energy funnel. This then traps the excitation at the outermost edge of the protein for subsequent transfer to nearby antennas or the thylakoid membrane-bound proteins. Interestingly though, ring D only has one close H-bond to a nearby Asp amide NH, with the pyrrolic NH nominally pointing toward the solvent interface. The other pyrrole rings are engaged in similar interactions to the other β-subunit bound bilins discussed above, with the propionic groups and central Asp providing significant anchor points to planarize the core.

In all cases, strong electrostatic interactions between each pyrrole ring with neighboring amino acids act to lock the full bilin structure in place in a rigid fashion. The ring D region of all bilins incorporated in the cryptophyte light-harvesting protein are firmly secured by a series of electrostatic interactions, and at times even covalent linkages (DBVs). This contrasts the relatively loose binding pocket typically observed in photosensory phytochromes and cyanobacteriochromes, where weaker and more mobile contacts (such as π−π stacking interactions with nearby aromatic residues) help facilitate structural changes between rings C and D following light absorption. This comparison, along with the disparate excited-state dynamics of the free subunits in solution, demonstrates the design principles utilized by photosynthetic organisms to foster specific molecular function. These insights gleaned from billions of years of evolutionary chemical design are crucial to the design and development of artificial, organic light-harvesting devices and photoswitching materials, which provide a cost-effective option for solar light harvesting applications.

## Data Availability

The raw data supporting the conclusions of this article will be made available by the authors, without undue reservation.
