# Supplementary material for: Spectroscopic and Photophysical Investigation of Model Dipyrroles Common to Bilins: Exploring Natural Design for Steering Torsion to Divergent Functions
Source: Front Chem. 2021 Feb 17;9:628852. doi: 10.3389/fchem.2021.628852 (PMC7925881; doi:10.3389/fchem.2021.628852)
Supplement: Supplementary file 1 [file table1.docx]

**Supplementary Material for:**

**Spectroscopic and Photophysical Investigation of Model Dipyrroles Common to Bilins: Exploring Natural Design for Steering Torsion to Divergent Functions**

Clayton F. Staheli,^1^ Jaxon Barney,^1,2^ Taime R. Clark,^1^ Maxwell Bowles,^1,3^ Bridger Jeppesen,^1^ Daniel G. Oblinsky,^4^ Mackay B. Steffensen,^1^ and Jacob C. Dean^1*^

**1. NMR Spectra of DPN and *N*-Me-DPN p. S2**

**2. DPN and *N*-Me-DPN dimer structures p. S4**

**3. Longer time DPN TA spectra p. S5**

**4. TA data for *N*-Me-DPN p. S5**

**5. TDDFT vertical excitation calculations and photoisomerization mechanism p. S7**

**1. NMR Spectra of DPN and *N*-Me-DPN**

**
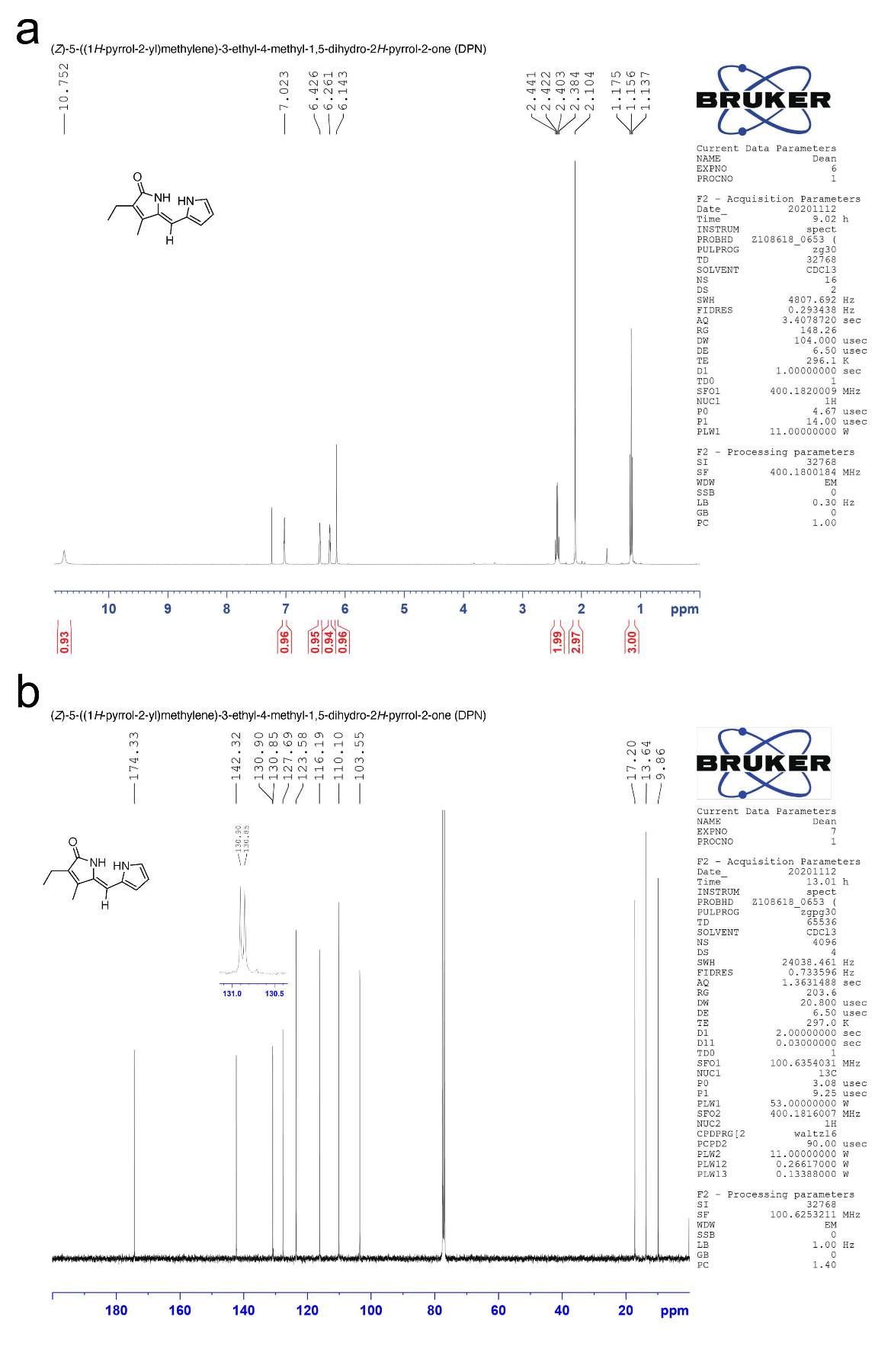
**

**
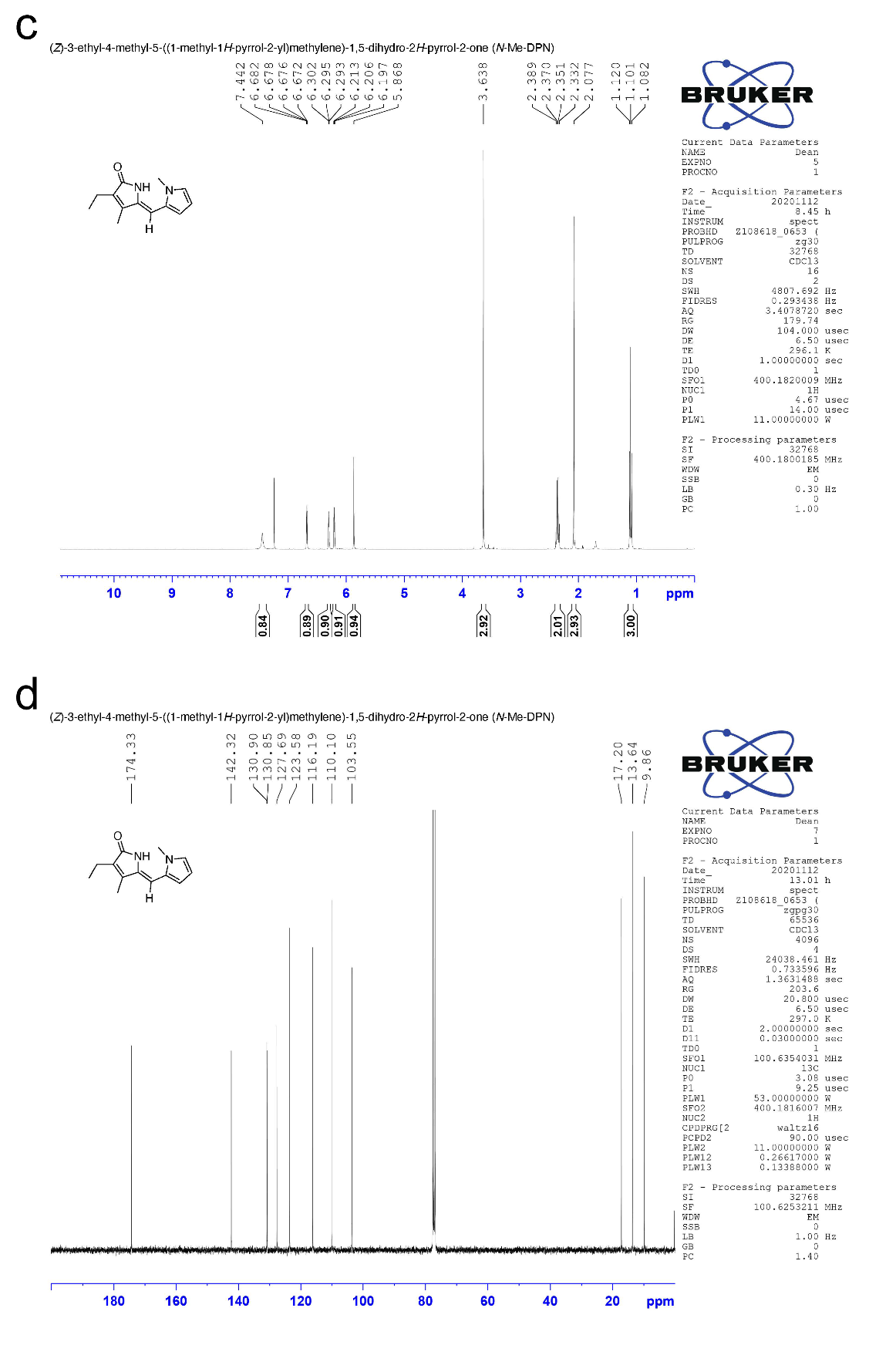
**

**Figure S1.** (a) Proton NMR and (b) carbon-13 NMR spectra for DPN synthesized for this work. (c) Proton NMR and (d) carbon-13 NMR spectra for *N*-Me-DPN synthesized for this work.

**2. DPN and *N*-Me-DPN dimer structures**

Hydrogen-bonded dimers of DPN readily form in nonpolar environments in a pseudo-*C*_2_ symmetric fashion wherein the pyrrolic NH groups both donate a H-bond to the carbonyl of the opposing DPN molecule. This leads to a total of four H-bonds spanning both molecules. As such, the (DPN)_2_ structure was calculated using the *Z*,*syn* configuration for each DPN monomer, and is shown in Figure S2a. With the addition of the bulky methyl group at the pyrrole NH position in *N*-Me-DPN, one full H-bond was lost from both monomers, yielding only two H-bonds bridging the dimer. In addition, the sterics associated with the methyl group significantly destabilizes the *Z*,*syn* dimer (Figure S2b, right) leading to a Gibbs energy ~200 kJ/mol larger than the *Z*,*anti* structure where the *N*-Me groups are oriented away from the opposing monomer (Figure S2b, left). The *Z,syn* structure forces the inter-ring angle of each monomer to ~40º in order to distance the methyl group from the opposing C=O group.

Comparing the binding free energy of the DPN dimer with that of the low energy *N*-Me-DPN (*Z*,*anti*) structure, we find a favorable Δ*G*_bind_ = −2.77 kJ/mol for DPN, versus +12.1 kJ/mol for the methylated case.


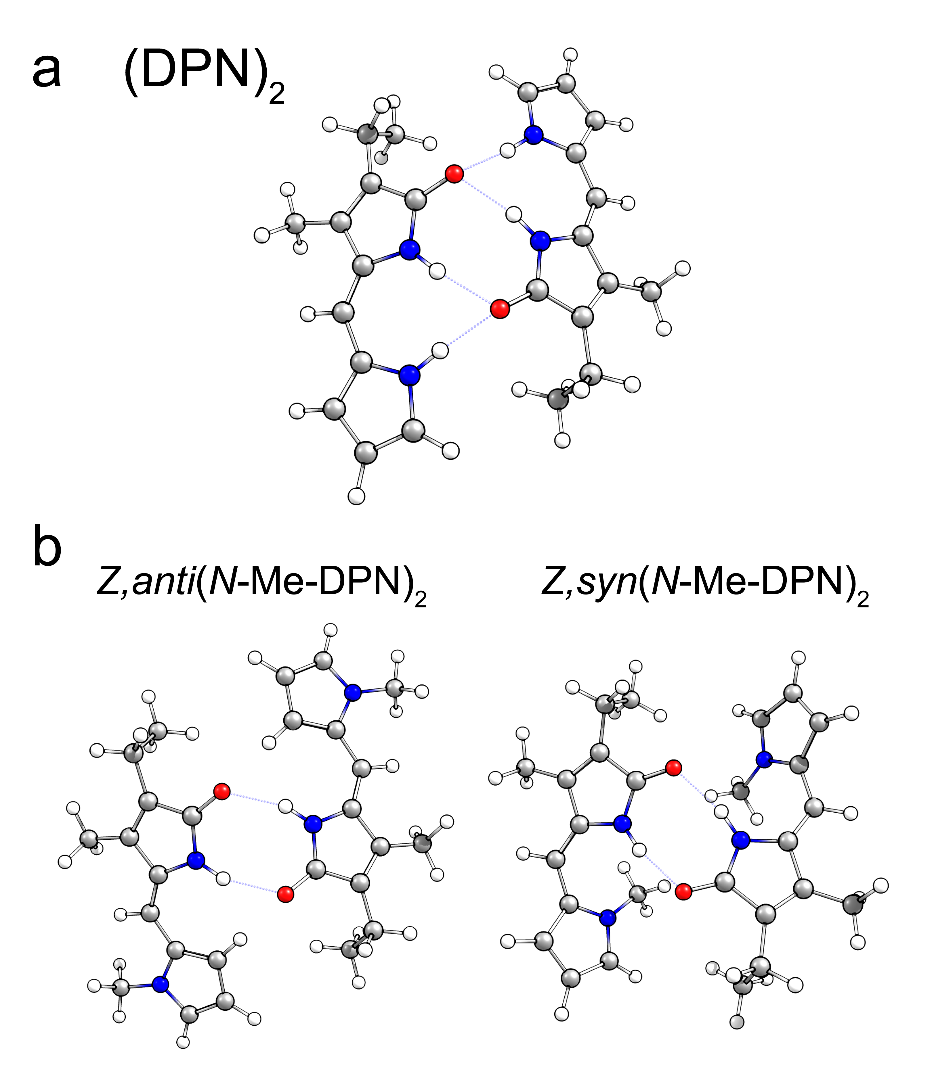


**Figure S2.** Dimer structures calculated for (a) DPN and (a) *N*-Me-DPN

**3. Longer time DPN TA spectra**

The progression of the primary GSB and red PIA features in DPN methanol TA data over ~100 ps reveals at least three independent populations relaxing with different kinetics. Figure S3a and S3b shows the TA spectra in the first 100 ps for DPN in methanol and dichloromethane respectively. Comparison of the longer time spectra shows this unique spectral shifting only in methanol solvent, suggesting conformational heterogeneity only in methanol as solvation about the DPN molecule breaks up the otherwise dominant (DPN)­_2_ population in nonpolar solvents.


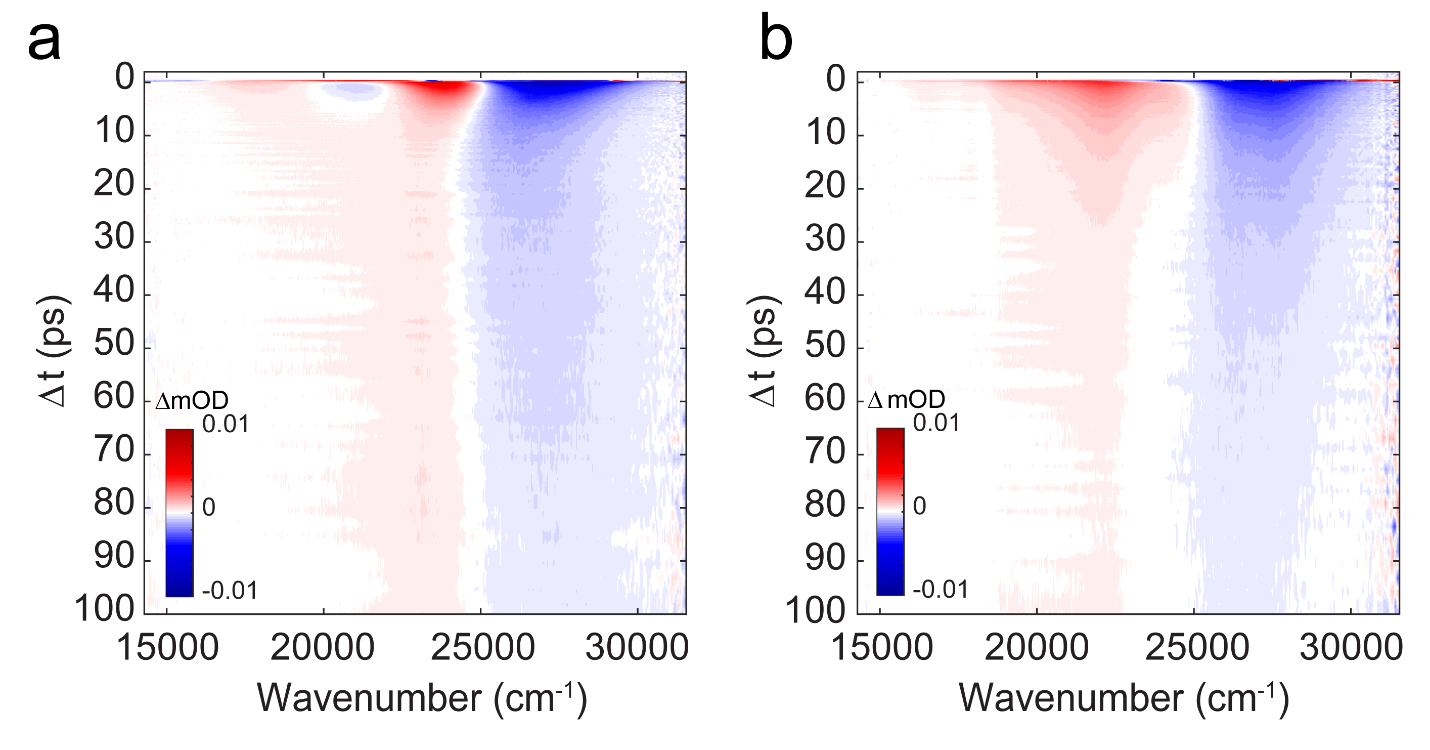


**Figure S3.** Transient absorption spectra of DPN in (a) methanol and (b) dichloromethane over the first 100 ps.

**4. TA data for *N*-Me-DPN**

In order to address the possible TA signatures of DPN dimer populations in solution, TA of *N*-methyl-DPN in methanol was performed. Those data are given in Figure S4, and are markedly similar to its DPN counterpart with the exception of a long-lived GSB feature present in DPN data but absent in *N*-Me-DPN. Interestingly, the ground state of *N*-methyl-DPN is completely re-filled by the end of the 200 ps experiment, and we can therefore tentatively associate the very small population remaining at long times in DPN data either to a dimer population or photoisomer.


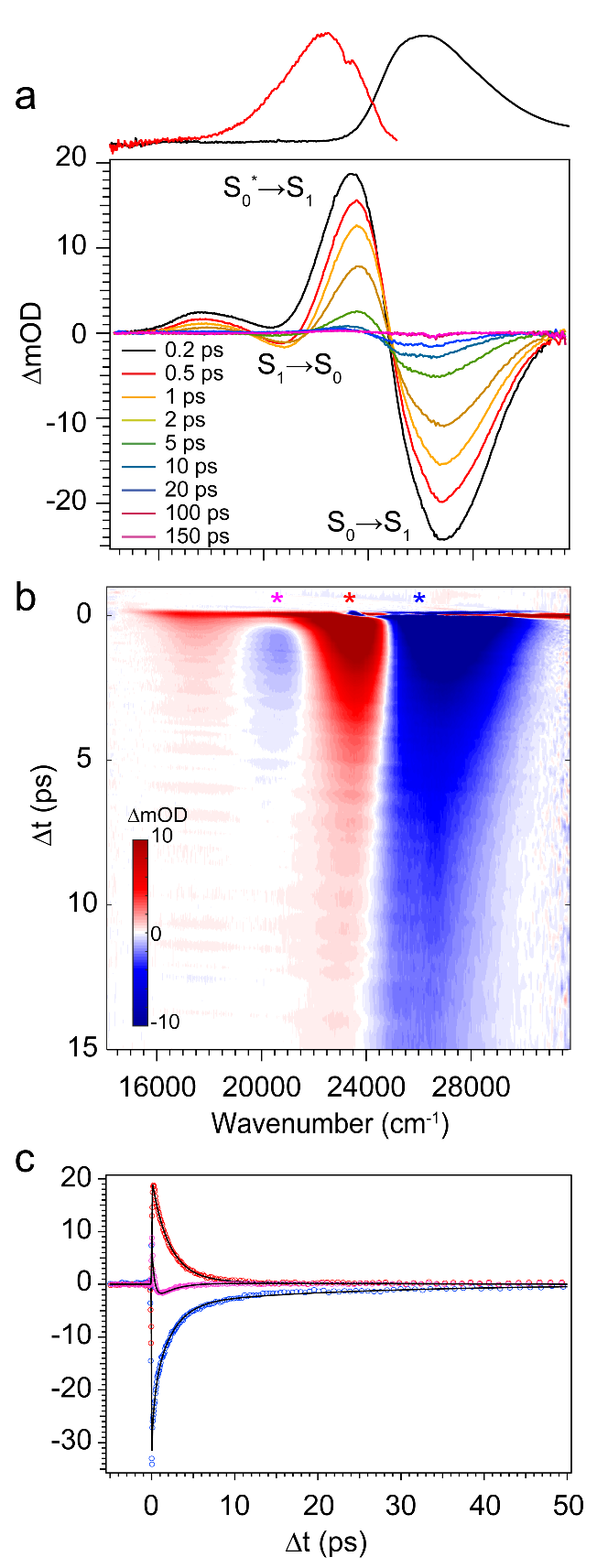


**Figure S4.** Transient absorption data for *N*-methyl-DPN in methanol. Asterisks in b denote wavenumber positions for transients shown in c.

**5. TDDFT vertical excitation calculations and photoisomerization mechanism**

The residual PIA signal just to the red of the DPY GSB remaining at the end of transient absorption experiments is assigned to a photoisomer generated from the small population that photoisomerizes about the inter-pyrrole methine bond(s). In order to make a tentative assignment to the conformer formed in the experiment, TDDFT vertical excitation energy calculations were performed to compare absorption positions of the strong S_0_-S_1_ transition. Table S1 shows the results for the *Z*,*Z*, *Z*,*E*, and *E*,*E* conformers calculated. According to these calculations, the *E*,*E* conformer uniquely absorbs just red of the primary *Z*,*Z* conformer by approximately 300 cm^-1^. Given the significant spectral overlap of the neighboring PIA and GSB signals, it is difficult to determine the exact wavenumber difference between these species; however, since the *E*,*E* species is the red-most absorbing of the three calculated we can assign the photoisomer on this basis. In this case, DPY undergoes a concerted twisting mechanism about both methine bonds linking the pyrrole rings to ultimately form the *E*,*E* configuration. The frontier orbitals associated with the HOMO→LUMO transition are shown in Figure S5, and the symmetric nature of the photo-induced anti-bonding character of both C_4_-C­_5_ and C_5_-C_6_­ bonds supports this hypothesis.

**Table S1.** Time-dependent density functional theory vertical excitation results calculated at the M05-2X/6-311++G(d,p) level of theory

|  |  |  |
| --- | --- | --- |
| **Conformer** | **Wavenumber (wavelength)** | ***f*** |
| *Z*,*Z* | 24703 (404.8 nm) | 0.9301 |
| *Z*,*E* | 25231 (396.3 nm) | 1.051 |
| *E*,*E* | 24400 (409.8 nm) | 0.9389 |

**
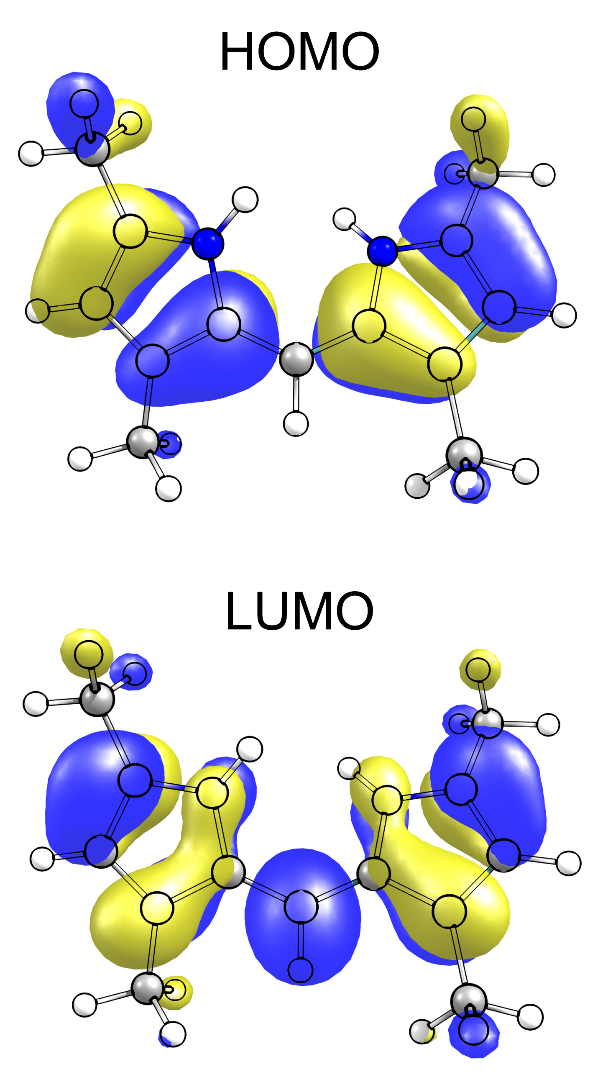
**

**Figure S5.** HOMO and LUMO orbitals associated with the S_0_–S_1_ transition of the primary *Z*,*Z* isomer of DPY
